# A Reinterpretation of Evidence for the Endothelial Glycocalyx Filtration Structure

**DOI:** 10.3389/fcell.2021.734661

**Published:** 2021-09-01

**Authors:** Kenton P. Arkill

**Affiliations:** School of Medicine, University of Nottingham Biodiscovery Institute, University Park, Nottingham, United Kingdom

**Keywords:** Starling hypothesis, vascular permeabilty, macromolecular transport, glycosaminoglycans, proteoglycan, heparan sulfate

## Abstract

The endothelial glycocalyx (eGlx) is thought to be the primary macromolecular filter for fluid flux out of the vasculature. This filter maintains the higher protein concentration within the vessel lumen relative to the tissue. Whilst the arguments for the eGlx being the size filter are convincing the structural evidence has been limited to specialized stains of perfusion fixed tissue, which are further processed for resin embedding for transmission electron microscopy. The staining and processing of the delicate pore structure has left many researchers struggling to interpret the observed surface coat. Previous work has alluded to a 19.5 nm spacing between fibers; however, whilst repeatable it does not give an eGlx pore size consistent with known glycosaminoglycan (GAG) molecular structure due to the required fiber thickness of >10 nm. Here a new interpretation is proposed based on the likelihood that the electron micrographs of are often of collapsed eGlx. The 19.5 nm spacing measured may therefore be the core protein of the proteoglycans (PGs) with the GAGs wrapped up around them rather than in an expanded *in vivo* state. The concept is explored to determine that this is indeed consistent with experimental measurements of permeability if the syndecans are predominately dimerized. Further an alteration of core protein lattice from hexagonal packing to square packing dramatically changes the permeability which could be facilitated *via* known mechanisms such as transient actin binding.

## Introduction

For most capillaries the primary method of fluid exchange between the plasma and the tissues is defined by the Starling Hypothesis as interpreted in 1997 by CC Michel (1997) and Weinbaum (1998) often dubbed the “revised Starling hypothesis.” With this interpretation, in the steady state, the oncotic pressure (Π) difference resisting the hydrostatic pressure (*P*) is across the endothelial glycocalyx (eGlx) rather than the whole vessel wall:

(1) $$J_{v}=L_{p}\,A\,\left[\left(P_{l\,u\,m\,e\,n}-P_{e\,G\,l\,x}\right)-\sigma \,\left(\Pi_{l\,u\,m\,e\,n}-\Pi_{e\,G\,l\,x}\right)\right]$$

Where the *J_v_* is the volumetric fluid flux, *L_p_* the hydraulic conductivity and *A* the capillary surface area. The osmotic reflection coefficient () (Staverman, 1951) is due to the membrane not being a perfect filter, and for albumin, the primary protein concentration in the plasma, the value is experimentally 0.9 ≤σ< 1. The fluid route out of the vessel is therefore *via* the eGlx, the endothelial junctions (or fenestrations), any significant basement membrane and finally between surrounding support cells (e.g., pericyte or podocyte). The role of the eGlx is to effectively block albumin, the primary oncotic pressure constituent, from leaving the vessel to equilibrate with the interstitum. The implication is that eGlx as a filter covers the whole intercellular junction entrance, so fluid is subsequently funneled into the junctions and further funneled through the junctional strands. The velocity becomes high enough from the funneling that albumin cannot diffuse against this, i.e., from the tissue to the vessel. If this is the case under steady flow across the vascular wall the oncotic pressure difference across the eGlx cannot be influenced by the interstitial albumin concentration. In Adamson et al. (2004) performed experiments which demonstrated this predication in single capillaries, whilst also mathematically predicting that the eGlx contribution to resistance to hydraulic flux is ≈50% in mesenteric capillaries. However; as later measured, the eGlx acts as a macromolecular filter, when chemically removed the hydraulic conductivity increased by 2.5-fold in line with an expected ≈50% increase but the albumin solute permeability increases by 20-fold (Betteridge et al., 2017). Perhaps it is fortuitous that the experimentally accessible mesenteric capillaries are relatively permeable. The cremaster muscle also accessible for such measurements has 10-fold more hydraulic resistance which may have made the signal-to-background too difficult to evaluate (Smaje et al., 1970). Of course, the different hydraulic conductivities across tissues are of physiological importance, and allow normal processes such as an immune response to manipulate the molecular size ratio of transported molecules (Owen-Woods et al., 2020).

Until this point the remaining unknown was to confirm the pore size of the eGlx. A study of electron micrographs and a limited number of Pt/C replicate micrographs from freeze-etched frog mesenteric and pulmonary vessels indicated a spacing between the eGlx fibers of 19.5 nm (Squire et al., 2001). Of note for this manuscript is that several different staining types gave the same measurement and this value also seen in follow up work on mammalian samples across stains in 2D and limited 3D quantification (Arkill et al., 2011, 2012, 2014). The space between fibers does not give us the size of the holes between the fibers and whilst some attempt a measurement was made in those works, this was clearly stain dependent and inconsistent. Fundamentally there is an issue between linking the ≈20 nm spacing repeatedly observed in perfusion fixed resin embedded electron microscopy and the expected 7–8 nm pore size to exclude albumin, as the required fiber thickness would be too large to fit with our understanding of eGlx composition. This manuscript explores a reinterpretation of what the ≈20 nm spacing represents, which whilst far from proof, leaves a hypothesis that fits links underlying biochemistry and maintains the observed physiology. Instead of the ≈20 nm spacing being the distance between fibers causing the filter, the spacing is the core protein that multiple glycosaminoglycan (GAG) side chains are attached to.

### Electron Microscopy of the Endothelial Glycocalyx

As discussed, the expected pore size to retain albumin in the lumen is around 7–8 nm diameter; therefore, the only method of visualization of this structure for the foreseeable future is transmission electron microscopy. An endothelial surface coat was first reported by Luft (1965). The difficulty in observation comes from a combination of technical challenges. Primarily, the physiological eGlx is dependent on plasma components, shear stress and other multiple cell type microenvironmental factors hencewith replication of the structure *in vitro* has been unsatisfactory. Secondly, to visualize the eGlx in resin sections one needs a counter stain.

Suitable stains include Alcian Blue 8GX (van den Berg et al., 2003; Betteridge et al., 2017) and similar cupromeronic blue (Meuwese et al., 2010), Ruthenium Red (Luft, 1966; Baldwin and Winlove, 1984), Cationised Ferritin (Clough, 1982), Tannic Acid (Qvortrup and Rostgaard, 1993), and lanthanides including lanthanum (Hjalmarsson et al., 2004; Chappell et al., 2009) terbium (Wagner and Chen, 1990), thorium (Hegermann et al., 2015), and mixtures such as LaDy (Arkill et al., 2012). Unfortunately, these electron dense stains are both non-specific and require perfusion fixation to get them to the eGlx. None of the standard stains prevent loss of the layer consistently. The favored reasoning being that there is an altered effect from shear stress when the eGlx is coated in stain. The final problem with all these stains is that they work by charge and therefore alter the structure (e.g., may collapse the layer) once it is coated, but even so the 19.5 nm spacing discussed occurs across these stains. Cryogenic immobilization techniques with freeze substitution have been attempted with various degrees of success (Ebong et al., 2011), although no direct visualization in amorphous ice has yet been observed.

### Composition of the Endothelial Glycocalyx Filter

The composition of the eGlx filter is thought to be the GAGs. All GAGs have a negative fixed charge density and the most notable for our case are heparan sulfate (HS), chondroitin sulfate (CS) bound in place by proteoglycans (PGs), and hyaluronan (HA) *via* CD44 if it is membrane bound. Of course, these GAGs have mechanistic roles and as such binding is dynamic (Smith et al., 2011; Dyer et al., 2016; Clausen et al., 2020). A GAG chain is a polysaccharide, in (very) simplistic terms a chain of carbon rings with side groups giving a diameter in the order of 1 nm. HS has been measured with molecular weights of 35–45kDa, although with overexpression of N-Deacetylase/N-Sulfotransferase-2 in HEK cells up to 160kDa (Lyon et al., 1994; Deligny et al., 2016) giving a chain length between 60 and 300 nm. The eGlx HS length has not been measured *in situ* in eGlx, but the 100–150 nm in length depicted is entirely feasible, whilst CS is a little longer than HS HA would typically be 250 nm to several mm in length (Cowman et al., 2015). Current dogma, which whilst likely has not been definitively shown, is that the eGlx filter itself is made from the sulfated GAGs and the HA forms a supportive role possibly under the sulfated GAG (Fan et al., 2019). The repeated observations of >1 μm thick eGlx indicate a multilayer structure and the full depth is unlikely to be the filtration mechanism. >1 μm thick, with a pore size of ≈7 nm would have a very low permeability not observed *in vivo*. The consensus is that the “filtration zone” is the membrane end of the eGlx, and bilayer implications on permeability have been explored (Curry and Michel, 2019). Perhaps noteworthy at this juncture is that the filter only needs to be over the intercellular junctions (in non-fenestrated vessels) and modeling suggests that this is only required to extend ≈200 nm away from the junction (Dalwadi et al., 2020).

There are therefore a multitude of unknowns and as such the subsequent interpretation of the electron microscopy is not claimed as definitive, but instead claims to fit current understanding consistently and therefore offers a promising alternative on previous interpretations.

## Materials and Methods

### Axioms

**FIGURE 1:**
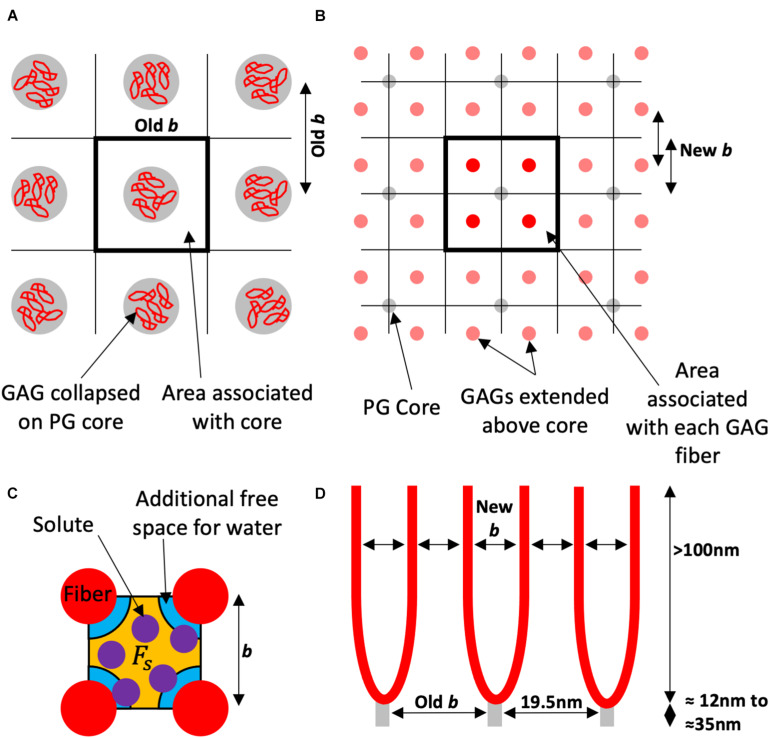
Schematic of the proposed model using the square lattice example. **(A)** Looking to the wall from the lumen the electron microscopy visualizes the proteoglycan (PG) core protein with the glycosaminoglycan (GAG) fibers collapsed around them. This gives an inter-fiber spacing (Old *b*) that has been measured experimentally as 19.5 nm, and an area associated with each fiber for the solvent and solute to pass. **(B)** The red GAG fibers (in this example four per core protein), are unraveled and spaced accordingly (in this case also into square ordering). Each fiber in this example has $\frac{1}{4}$ of the associated area to the PG core protein, and a new spacing (New *b*). **(C)** The orange free space (*F*_*s*_) for the purple solute is a proportion of the free space for water which has the orange and the additional blue space. The water is only excluded by the fiber diameter. This proportion is used to estimate the solute reflection coefficient. **(D)** Is a side view of the endothelial glycocalyx (eGlx) with the PG core protein spaced as “Old *b*” or 19.5 nm. There is now a new spacing “New *b*” between GAG fibers and a different fiber diameter to calculate the proportions of free space.

Here it is explored that the filter is determined from the sulfated GAGs perpendicular to the membrane extending over 100 nm into the lumen, and that the 19.5 nm spacing observed is the core protein spacing not the fiber spacing thought previously (Figure 1). These core proteins have the GAG chains attached and they will therefore find an equilibrium spacing, effectively making the membrane area covered by a single core protein the area covered by the separated multiple GAG fibers attached to that protein core.

### Estimates and Variables

The reflection coefficient can be estimated or predicted from a structure. Here, the derivation of Anderson and Malone (1974) is used, which is applicable to long pores with a spherical non-binding solute:

(2) $$\sigma ={\left(1-\emptyset \right)}^{2}$$

Where ∅ is the partition coefficient defined as the relative free space between the solute (*F_s_*) and the water (*F_w_*):

(3) $$\emptyset =\frac{F_{s}}{F_{w}}$$

The partition coefficient can be calculated by geometry (Figure 1C) as previously for hexagonal packing (Zhang et al., 2006) and for a square lattice (Arkill et al., 2011). In this case the supplementary material from Arkill et al. (2011) is used, as contains the derivations for both lattices. The albumin’s Stoke–Einstein radius as deemed to be 3.5 nm.

Fiber Diameter has been a major problem with interpreting the spacing data with estimates from the electron dense staining ranging from 3 nm up to 18 nm. As discussed in Arkill et al. (2011) the measured sizes anecdotally tend to rise in 3 nm jumps consistent with a stain such as Alcian Blue coating a ≈1 nm fiber and then clumping together. Regardless, clearly the stains are affecting the structure, and of course adding to the size of the GAGs when bound. Here, we estimate for a range of fiber diameters ranging from 0.5 to 3 nm depicted as an error on the varied parameter. The expectation is that the true diameter is around 0.4–1 nm (Ogston and Preston, 1966); however, the Debye length (Debye and Hückel, 1923) for charge shielding is *circa* 0.5–1 nm, which therefore could effectively add up to 2 nm to the diameter of each fiber for negatively charged molecules.

Number of Fibers per core is also uncertain. Transmembrane Syndecan-2 and Syndecan-4 have three GAG attachment sites, whereas Syndecan-1 has five and Syndecan-3 has six, though the latter has limited presence in endothelium (Couchman et al., 2015). Syndecans are also expected to occur in dimers although other configurations exist (Godmann et al., 2020; Chen et al., 2021). Glypicans, likely more mobile in the outer layer only of the membrane lipid bilayer, have two GAG chains are also abundant in the endothelium (Iozzo and Schaefer, 2015).

Fiber Spacing has been measured as 19.5 nm (Squire et al., 2001; Arkill et al., 2011; Figure 1A), but if we factor in section shrinkage from electron flux can be 8–10% (Mantell et al., 2012) in x-y orientations and general variation between samples we have displayed from 13 to 25 nm. There is likely a periodic core structure which can be square, possibly due to interactions of transmembrane syndecans with the actin cytoskeleton or a less constrained hexagonally spaced. As the multiple GAG chains are expanded off the PG core to fill the same area they too can be square or hexagonally packed. Here we display Hexagonal core with Hexagonal GAGs (Hex-Hex), Square core with Hexagonal GAGs (SQ-Hex), and Square core with Square GAGs (SQ-SQ). The latter is intuitively unlikely unless there are other regular supports in the structure (examples could include albumin or possibly HA).

## Results and Discussion

The SQ-SQ has a more open structure than Hex-Hex for equivalent fiber parameters, perhaps less intuitively the SQ-Hex is more open than SQ-SQ structures as in the latter the GAG chains’ nearest neighbor is forced closer and is more constrained.

### Dependance on Number of Fibers per Core

**FIGURE 2:**
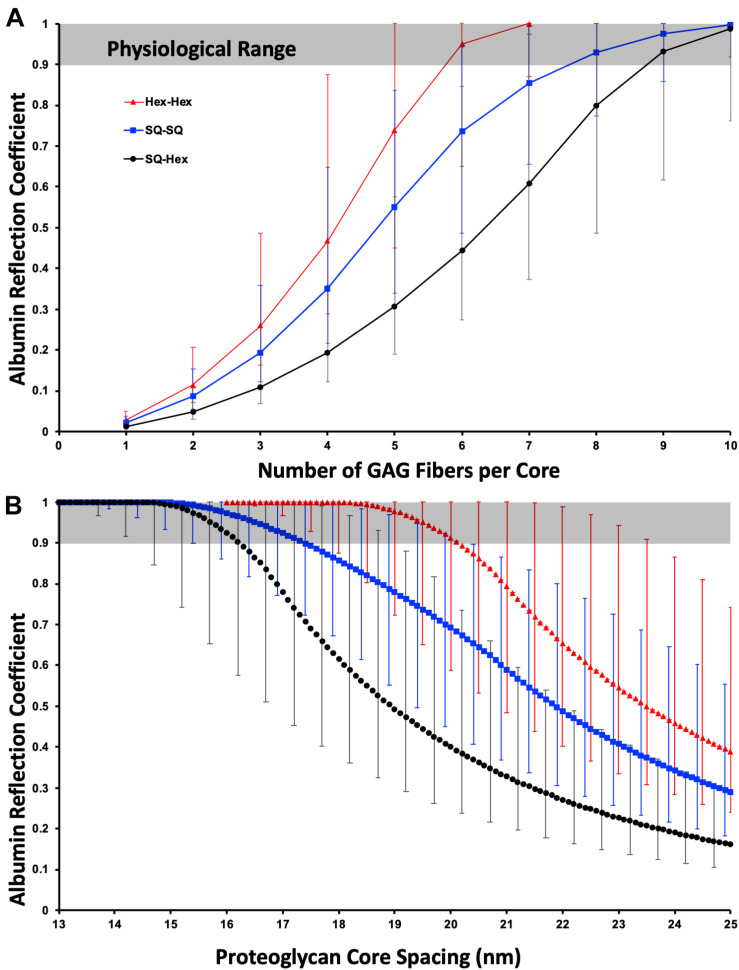
The physiological albumin reflection coefficient fits well with a hexagonally packed dimerized PG core with a total of ≥6 GAG chains with a diameter of 1.5 nm (including charge effects). **(A)** Variation of reflection coefficient with the number of GAG chains per PG core structure. The data is depicted for hexagonal and square core organization (Hex- and SQ-, respectively) with the overlaying GAG chain organization hexagonal (Hex) or square (SQ). **(B)** As A but demonstrating the dependance of reflection coefficient on fiber spacing with six GAG chains per core structure. The physiological reflection coefficient is the grayed area. The error bars are for a 0.5 nm (lower reflection coefficient) and a 3 nm (higher reflection coefficient) fiber diameter.

Figure 2A illustrates that for 19.5 nm core spacing the proposed model is only consistent with physiological experiments (0.9 ≤ σ < 1) when there are at least five, but almost certainly at least six GAG chains; therefore, the estimate that there are likely six from syndecan-2 and -4 in dimers seems to hold well. There are likely to be mixtures of syndecans present and so whilst only dimerized syndecan-1 would exclude albumin, raising the mean fiber number to seven or even eight GAGs per core is still possible. Clearly for this model to be true the syndecans must dimerise in normo-physiology.

### Dependence on the Spacing Value

Figure 2B illustrates that for six GAG chains per core is highly sensitive to the fiber spacing. Of note, and perhaps what spurred the dissemination of this work, is that for a 1.5 nm diameter fiber, the most justifiable from the addition of native core and Debye shielding diameter, for albumin falls in the physiological range for Hex-Hex for an inter-fiber spacing between 18.2 and 20 nm. A slightly larger spacing would require either a slightly thicker GAG fiber or more GAG chains per core, both options are likely. SQ-SQ, without further structural evidence, makes it the least likely formation, as it would need more GAG chains or the extreme end of the fiber thickness estimate to be consistent with the model. SQ-Hex formation is almost certainly not the formation in normo-physiology.

### Strengths and Weakness of the Proposed Filter Model

Here it is proposed that the filter model which previously measured inter-fiber spacing of 19.5 nm by transmission electron microscopy is false and that this spacing is instead the inter-PG core spacing with the GAG chains collapsed around them. These calculations show that the proposed model fits decades of both *in vivo* experimental measurements and our (limited) biomolecular understanding of the sulfated GAGs in the eGlx. There are two particular additional strengths for the proposed model:

Firstly, that as the GAG chains are floating above the core protein, and likely self-spacing, the system has built-in flexibility missing from the original model. One can envisage a missing core protein (or GAG chain) due to engagement in a mechanistic role or simply random chance, and the remaining GAG chains drifting slightly further apart, but still performing their albumin excluding role. Thus, the proposed model is flexible enough to allow for physiological variation in an essential system, and hopefully pacify some valid concerns especially with ignored variables such as membrane curvature around fenestrations and intercellular junctions or the unlikely rigidity of spacings as modeled here.

The second is that a change of lattice structure, for example to a SQ core formation, would radically alter the reflection coefficient to albumin and indeed the permeability to macromolecules. Whilst speculative in the context here, this type of formational change is feasible, for example by selective binding mechanisms to the actin cytoskeleton (Yoneda and Couchman, 2003; Multhaupt et al., 2009; Li and Wang, 2019), and would allow for great functional control over what is a dynamic system. Further, there would still be direct GAG control over the filtration *via* other mechanisms, such as those hypothesized in immunology for clumping of the HS leaving short term gaps (Dyer et al., 2017; Handel and Dyer, 2021) as well as the activity of shedases [e.g., (Annecke et al., 2011)].

Weaknesses in the new filter model stem from a current lack of biomolecular knowledge, particularly *in vivo*, which limits our interpretation substantially. What is the physiological composition of the eGlx? Are components there by chance, transiently, or do they have a structural role? Perhaps the largest structural unknown is that of HA. It is abundant and tends to have a structural role, but is the HA located amongst the sulfated GAG, running underneath or looping over the top? Is the HA involved in determining the pore size and organization directly or indirectly? The model does not include the outer layer of the eGlx that seems to exist, which perhaps is the layer now dubbed “perfused boundary region” in human *in vivo* detection (Nieuwdorp et al., 2008). Certainly, the size of the eGlx has been robustly measured in the 1 μm range, and this is far too thick to account for the observed permeability if it is all “filtration zone.” There also remains the question of the outer region’s composition as sulfated GAGs are not expected to be that long. There are also longer spacings noted by Squire et al. (2001), Arkill et al. (2011), and Hegermann et al. (2015). These do not seem to fit a multiple of the 19.5 nm spacing, but are around 50 nm. There is no evidence on what these are, however, convenient it would be for them to be HA membrane binding. A fuller understanding of the biochemistry of the filter would inform the validity of many of the mathematical assumptions and approximations here. Perhaps the main two being the validity of Eq. 2 (that requires long pores with a spherical non-binding solute), and the distribution of fiber spacings only as a mean distance is considered not the variance in distribution around that mean.

Direct visualization of the structure, is technically very challenging. To visualize the eGlx *in situ* by cryogenic TEM may be possible with the emergence of direct electron cameras and phase plates becoming more accessible. Continuing freeze-etching techniques (Squire et al., 2001; Sun et al., 2020) on eGlx are another method worth pursuing. Both these methods would benefit from correlating with optical fluorescence to be sure of imaging location post tissue fracture, and unfortunately higher throughput. The proposed model whilst not definitive would allow for biomolecular testing that does not rely on direct visualization, such as syndecan ratios or GAG chain length, that perhaps will allude to the full eGlx structure. Once such possibility imaging mass spectrometry that can separate GAG composition (Hook et al., 2021) and can have <100 nm precision. Further the model can indirectly be compared to others by determining the reflection coefficient (along with the permeability) on the same vessel from multiple charged and sized solutes, such as organic nanodots (Mongin et al., 2008) as $\frac{F_{s}}{F_{w}}$ will vary in a unique manor.

## Conclusion

The model proposed here fits both the historical electron microscopy and our current understanding of the sulfated GAGs in the eGlx. It also highlights that a change in formation, for example *via* actin binding, would dramatically alter the size of macromolecule that can pass through to the junction or the membrane.

## Data Availability Statement

The original contributions presented in the study are included in the article/supplementary material, further inquiries can be directed to the corresponding author.

## Author Contributions

The author confirms being the sole contributor of this work and has approved it for publication.

## Conflict of Interest

The author declares that the research was conducted in the absence of any commercial or financial relationships that could be construed as a potential conflict of interest. The reviewer FC declared a past co-authorship with the author to the handling editor.

## Publisher’s Note

All claims expressed in this article are solely those of the authors and do not necessarily represent those of their affiliated organizations, or those of the publisher, the editors and the reviewers. Any product that may be evaluated in this article, or claim that may be made by its manufacturer, is not guaranteed or endorsed by the publisher.
